# Mitochondrial DNA Haplotypes Influence Energy Metabolism across Chicken Transmitochondrial Cybrids

**DOI:** 10.3390/genes11010100

**Published:** 2020-01-16

**Authors:** Minghua Kong, Hai Xiang, Jikun Wang, Jian Liu, Xiben Zhang, Xingbo Zhao

**Affiliations:** 1 National Engineering Laboratory for Animal Breeding; Key Laboratory of Animal Genetics, Breeding and Reproduction, Ministry of Agriculture; College of Animal Science and Technology, China Agricultural University, Beijing 100193, China; kmh928335059@163.com; 2 Guangdong Provincial Key Laboratory of Animal Molecular Design and Precise Breeding, School of Life Science and Engineering, Foshan University, Foshan 528225, China; vamyluo@126.com; 3 Key Laboratory of Qinghai-Tibetan Plateau Animal Genetic Resource Reservation and Utilization, Sichuan Province and Ministry of Education, Southwest Minzu University, Chengdu 610041, China; xdzwjk@163.com; 4 Guizhou Nayong Professor Workstation, Bijie 553300, China; liu123985442@126.com (J.L.); 13308579937@163.com (X.Z.); 5 Institute of Animal Husbandry and Veterinary Medicine, Bijie 551700, China

**Keywords:** mitochondrial DNA haplotype, cybrids, energy metabolism, chicken breed

## Abstract

The association between mitochondrial DNA haplotype and productive performances has been widely reported in chicken breeds. However, there has not been physiological evidence of this seen previously. In this study, chicken transmitochondrial cells were generated using the nucleus of the DF-1 cell line and mitochondria of primary cell lines derived from two native chicken breeds, Tibetan chicken and Shouguang chicken. Generally, Tibetan chicken primary cells showed a stronger metabolic capacity than Shouguang chicken primary cells. However, the Tibetan chicken cybrids had a dramatic drop in relative mtDNA copies and oxygen consumption. Higher rates of oxygen consumption (OCR) and expression levels of mitochondrial biogenesis and fusion genes were observed in Shouguang chicken cybrids, potentially reflecting that the mitochondrial DNA haplotype of Shouguang chicken had better coordination with the DF-1 nucleus than others. Meanwhile, mitonuclear incompatibility occurred in Tibetan chicken cybrids. The results demonstrate functional differences among mitochondrial DNA haplotypes and may shed light on the interaction between the mitochondria and nucleus in *Gallus gallus domesticus*.

## 1. Introduction

The mitochondrion occupies a crucial position in almost all multicellular organisms, principally involved in the conversion of energy through respiratory chains [1]. In addition to cellular energy production, the mitochondrion houses essential pathways of heme synthesis, signal transduction, and apoptosis [2]. 

For millions of years, mitochondrial DNA (mtDNA) haplotypes have evolved, and certain mtDNA variants have been inherited [3]. Not only do mtDNA haplotypes vary among breeds and strains, but they confer positive and negative advantages to the organisms. For instance, mtDNA haplotypes influence the rate of radiographic OA progression and encephalomyopathies, adaptation to cold environments, and are responsible for the complex phenotypes of aging and longevity [3,4,5,6]. For animals, mtDNA haplotypes have been reported to associate with economically important traits, such as the milk quality of Holstein cows and reproductive capacity of pigs [7,8]. Many studies also indicate that mtDNA polymorphisms are correlated with growth traits, such as pectoral muscle fat content, duodenum length, Marek’s disease resistance, and hypoxia adaptation in chicken breeds [9,10,11]. 

However, due to significant differences in the nucleus, environmental factors, and epigenetic modification, it has been challenging to evaluate the effect of the specific mtDNA haplotype on complex traits [12]. Moreover, the central point in defining the role of mitochondria in complex traits is to comprehensively understand the relationship between the nuclear genome and the mtDNA haplotype. At the organismal level, conplastic animals were generated by backcrossing females (mitochondrial donors) with males (nuclear donors) for over 20 generations; then, complex traits were studied, such as longevity in conplastic mice and fitness in conplastic *Drosophila* [13,14]. In order to study the genetic and metabolic consequences caused by different mtDNA haplotypes at the cellular level, cytoplasmic hybrids (cybrids) are created, where the mitochondria donor cells are enucleated and then transfer to the nuclear donor cells previously depleted of their own mtDNA. The cybrids generated through this strategy maintain the constant nuclear components, so the cellular changes caused by the transplanted mtDNA can be studied [15,16].

China is a native country of chicken production, with a long cultivation history and abundant resources. Compared with commercial breeds, native chickens have many desirable characteristics, such as suitability to environments (hypoxic adaptation), disease resistance, and excellent meat quality. The Tibetan chicken is a native breed that has inhabited the Qinghai–Tibet Plateau (4000 m above sea level on average) for thousands of years and has adapted well to the harsh environments, high altitude, hypoxia, and cold conditions [17]. Tibetan chickens are widely used in agricultural practices, providing local people with meat and eggs. However, egg and meat production of Tibetan chicken is much lower than the breed which lives in the plain, such as the Shouguang chicken, which has a significant advantage in body growth and egg production. Animal products, such as meat and eggs, can be considered as a transformation from chemical energy through mitochondrial metabolism [18].

In this study, we chose the DF-1 cell line [19] as the nuclear donor, and primary embryo fibroblast cell lines from Tibetan and Shouguang chicken as mitochondria donors, to generate cybrid cells. These cybrids harboring different mtDNA haplotypes displayed variable expressions of genes associated with mitochondrial biogenesis and mitochondrial fusion. Most importantly, they presented significant differences in energy metabolism. Our study is the first to identify the effect of mtDNA haplotype on energy metabolism in Chinese native chicken breeds by using transmitochondrial cells.

## 2. Materials and Methods

### 2.1. Cells Preparation and Culture Conditions 

All the experimental procedures followed the guidelines for animal management of China Agricultural University (CAU), and the experimental protocols were allowed by the Experimental Animal Care and Use Committee of CAU (approval code: CAU20151205-5).

The chicken embryo fibroblast cells (DF-1) from the East Lansing Line were gifted by Professor Zhao Yaofeng, China Agricultural University. Primary chicken embryo fibroblasts were isolated from two native chicken breeds, T from Tibetan chicken and S from Shouguang chicken. The growth medium used was DMEM supplemented with 10% FBS, 100 µg/mL streptomycin, and 100 U/mL penicillin. For ρ^0^ cells, an extra 50 µg/mL uridine was added into the medium. All the cells were cultured in 5% CO_2_/95% air at 37 °C.

### 2.2. Generation of Cybrids

Using Lipofectamine 2000 (Invitrogen, Carlsbad, CA, USA), plasmid p-eGFP-neo was transfected into DF-1 cells (D), which was used as a selection marker for successful cybrids. ρ^0^ cells and enucleated mitochondrial donor cells were created according to the modified procedures of Bacman and Moraes [20]. DF-1 cells, the nuclear donor cell line (ρ^0^), were cultured with 2.5 µg/mL rhodamine 6G (R-6G) for five days. The other primary embryo cells (T and S) were used as mitochondria donor cells. Cybrids were created by the fusion of the ρ^0^ cells with the enucleated cells using polyethylene glycol (PEG) [20,21].

### 2.3. Mitogenome Sequencing 

A total of 22 pairs of PCR primers were used to amplify the whole chicken mtDNA (Supplementary Table S1). Sequences were analyzed and submitted to GenBank. The polymorphisms were compared using MEGA6. The conservation of the missense mutations was evaluated by the Grantham Score [22,23,24].

### 2.4. Metabolic Assays

The specific medium for the mitochondrial stress test and glycolysis test was made, respectively. For the mitochondrial stress test, unbuffered DMEM supplemented with 2 mM sodium pyruvate, 10 mM D-glucose, and 4 mM L-glutamine was used, and the pH was adjusted to 7.4. For the glycolysis test, unbuffered glucose-free DMEM containing 4 mM L-glutamine was used, and the pH was adjusted to 7.35. 

The cells were cultured in 6 wells of XF96 cell culture microplates (Seahorse Bioscience) at the density of 1.8 × 10^4^ cells per well. The sensor cartridge was hydrated by adding 200 μL of XF calibrant solution to each well, and incubated in a 37 °C, CO_2_-free incubator overnight. The next day, the normal cell medium was replaced with the assay medium. For the mitochondrial stress test, reagents were injected by following the protocol of the Seahorse XF Cell Mito Stress Test kit. First, the “resting” OCR was established, and then oligomycin (2.0 μM) was added. Oligomycin inhibits complex V (ATP synthesis), so the difference before and after oligomycin can be calculated as the OCR linked to ATP production. Subsequent injection of FCCP (0.25 µM), which is an uncoupler, was used to indicate the maximum rate of respiration that the cell can achieve. Finally, rotenone (1 µM) and antimycin (1 µM) were added to inhibit complex I and complex III to shut down the respiration chain. Thus, non-mitochondrial respiration can be calculated. For glycolysis analyses, reagents were injected by following the protocol of the Seahorse Glycolysis Stress Test kit. First, glucose (10 mM) was added. Then, oligomycin (2 µM) was injected to establish the “highest” ECAR by shifting the energy generation to glycolysis. The last injection is 2-DG (100 mM)) measured the non-glycolytic ECAR. The results were normalized by the CyQUANT Cell proliferation kit (Invitrogen). OCR was shown as pmol O_2_/min/1.8 × 10^4^ cells; ECAR was shown as mpH/min/1.8 × 10^4^ cells.

### 2.5. Total DNA Isolation and Relative mtDNA Copies Detecting

High-quality genomic DNA was extracted from all the cell lines and the relative mtDNA copies were determined using real-time PCR [25]. A pair of primers (Forward 5′—3′: CCAACCACCAACCTGATAGC; Reverse 5′—3′: TGTGGGATGGAAGAGTGCC) was designed to target the ND4 of mtDNA (KM433666.1). The 18S rRNA gene was used for standardization (Forward 5′—3′: ATAACGAACGAGACTCTGGCA 3′; Reverse 5′—3′: CGGACATCTAAGGGCATCACA). The optimum thermal cycling parameters were 95 °C for 15 min, 40 cycles of 95 °C for 10 s, 58 °C for 20 s, and 72 °C for 25 s. The lengths of the amplification product were 157 bp and 136 bp, respectively. The cycle threshold (C_T_) was used for calculations. The ΔC_T_ was calculated by the subtraction of the average 18S rRNA C_T_ value from the average ND4 C_T_ value. We used the cybrid D+D to normalize and subtracted the ΔC_T_ of D+D from the ΔC_T_ of each group, yielding the value of ΔΔC_T_. The fold change of relative mtDNA copies were detected with the 2^−ΔΔCT^ method [26].

### 2.6. Total RNA Extraction and qPCR

Total RNA was extracted from all the cell lines by using the RNeasy Mini Extraction (Qiagen) and RNase-Free DNase Set (Qiagen). Then 500 ng of RNA was taken to do reverse transcription by using the QuantiTect Reverse Transcription Kit (Qiagen) for Q-PCR analyses. The SYBR Green PCR Master Mix (Tiangen) and Bio-Rad iCycler iQ5 detection system were used to perform the Q-PCRs. The cybrid D+D was used as the standard, and the expression levels were calculated with reference to expression of *β-actin*. The results were shown as a fold change using the 2^−ΔΔCT^ method [26]. The sequences of all the primers were listed in Supplementary Table S2. 

### 2.7. Statistical Analysis

Statistical analyses were performed using SPSS 20.0 software (SPSS Inc., Chicago, IL, USA). Tukey’s multiple comparison test of ANOVA was used to detect the main effect of the mtDNA haplotypes. Data are exhibited as means ± SEM.

## 3. Results

### 3.1. Generation of Cybrids

**Figure 1:**
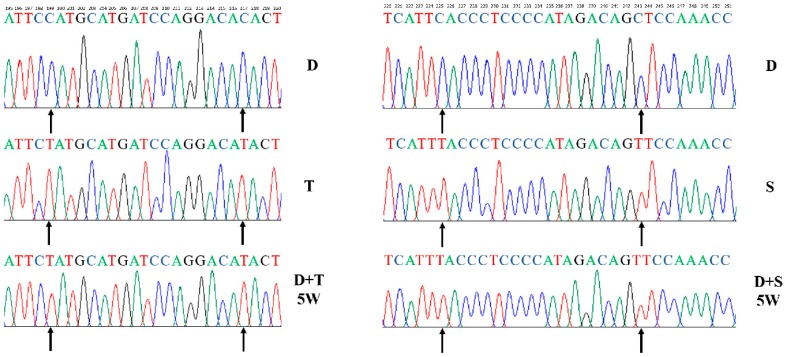
Sequence verification in the generation of different cybrid cells. D, T, and S harbor characteristic sequence signatures within the D-loop region. The endogenous mtDNAs (D) were no longer detectable in the fifth week (D+T/5W, D+S/5W). In cybrid cells, the acronym D denotes a common nucleus of DF-1, and +T and +S represent the source of the mitochondria. 5W: cell culture for five weeks.

DF-1 fibroblast cells (D) were cultured in medium containing rhodamine 6G (R-6G) to create ρ^0^ cells. Primary embryo fibroblast cells from Tibetan chicken (T) and Shouguang chicken (S) were used as mitochondrial donor cells. We found that endogenous mtDNA was substituted for exogenous mtDNA completely by PCR-amplifying the mtDNA control regions after clone selection. Results showed that cybrids (D+T, D+S) only contained the Tibetan chicken (T) and Shouguang chicken (S) mtDNA, respectively. The endogenous mtDNA (D) cannot be detected after five weeks (Figure 1).

### 3.2. Mitogenome Sequencing 

The mtDNA haplotypes of all the chicken cell lines (D, T, and S) were sequenced and submitted to GenBank with accession numbers (MK163561–MK163563). After determining the mtDNA sequence of the cybrids (D+D, D+T, and D+S), we found that the mtDNA sequences of each cybrid cell were identical to the mitochondrial donor cells (Figure 1).

The polymorphisms of the three mtDNA haplotypes were summarized in Table 1. Specifically, the alignment T/S harbored 41 variations, while D/T and D/S presented 39 and 46 variations, respectively. T/S showed 25 mutations in the 13 mtDNA peptide-coding genes, including five missense mutations, of which one had conservative Grantham scores and four had moderately conservative Grantham scores [22,23]. D/T and D/S presented five and four missense mutations, respectively. More details of the polymorphisms were listed in Supplementary Table S3 and S4.

### 3.3. Metabolic Assays

The Seahorse XF96 Analyzer was used to quantify oxidative phosphorylation (OXPHOS) and glycolysis of chicken primary cells and cybrid cells. Following the protocol with specific components, the level of OXPHOS and glycolysis was evaluated by the mitochondrial stress test and glycolysis stress test, respectively. 

**Figure 2:**
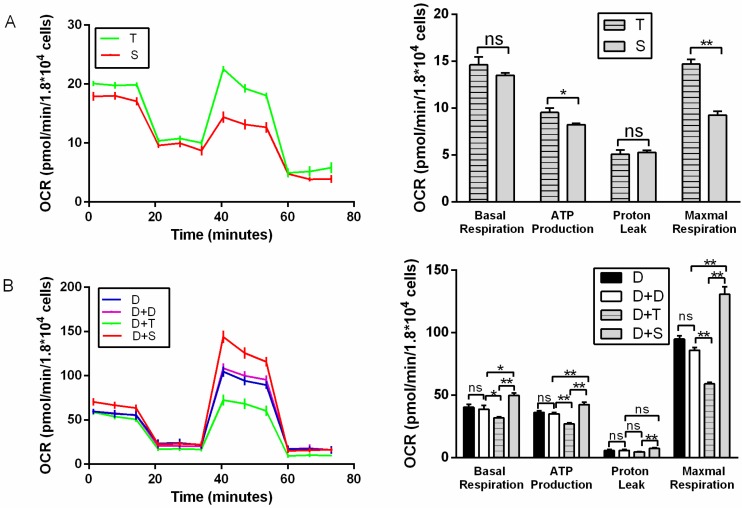
Mitochondrial stress test for primary chicken cells and cybrids. (**A**) Illustrating OCR of primary cell lines (T, S). (**B**) Illustrating OCR of cybrid cells; D was used as a control for D+D. Oligomycin, FCCP, and rotenone plus antimycin A were injected sequentially to the cells. OCR profiles were expressed as pmole O2/min/1.8 × 10^4^ cells. In cybrid cells, the acronym D denotes a common nucleus of DF-1 cell, and +T and +S represent the source of mitochondria. *n* = 6 per group. * means *p* < 0.05, ** means *p* < 0.01, and ns means *p* > 0.05.

**Figure 3:**
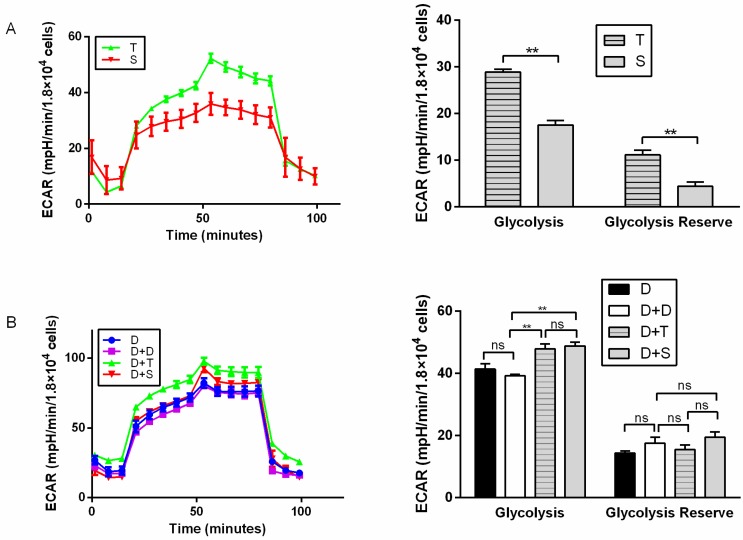
Glycolysis test for primary chicken cells and cybrids. (**A**) Illustrating ECAR of primary cell lines (T and S). (**B**) Illustrating ECAR of cybrids, D was used as a control for D+D. All cells exposed sequentially to glucose, oligomycin, and 2-DG. ECAR profiles were expressed as mpH/min/1.8 × 10^4^ cells. *n* = 6 per group. ** means *p* < 0.01 and ns means *p* > 0.05.

For primary cells, T generally showed higher basal respiration, ATP production (*p* < 0.05), and maximal respiration than S (Figure 2A). T also showed higher ECAR than S, with higher glycolysis and glycolytic reserve (*p* < 0.01) (Figure 3A). It indicated that Tibetan chicken cells have a higher rate of anaerobic respiration due to their environment. In contrast, the Tibetan chicken cybrid (D+T) showed lower OCRs than the Shouguang chicken cybrid, with lower basal respiration (*p* < 0.01), ATP production (*p* < 0.01), and maximal respiration (*p* < 0.01) (Figure 2B). These results indicated the inferior capacity in mitochondrial function and energy metabolism in the Tibetan chicken cybrid. The Shouguang chicken cybrid (D+S) also presented a higher OCR than D+D, with higher basal respiration (*p* < 0.05), ATP production (*p* < 0.01), and maximal respiration (*p* < 0.01) (Figure 2B), demonstrating a significant increase in energy metabolism. The results above can allow us to measure the metabolic properties of the chicken primary cells and cybrid cells.

### 3.4. Relative mtDNA Copies Detecting

**Figure 4:**
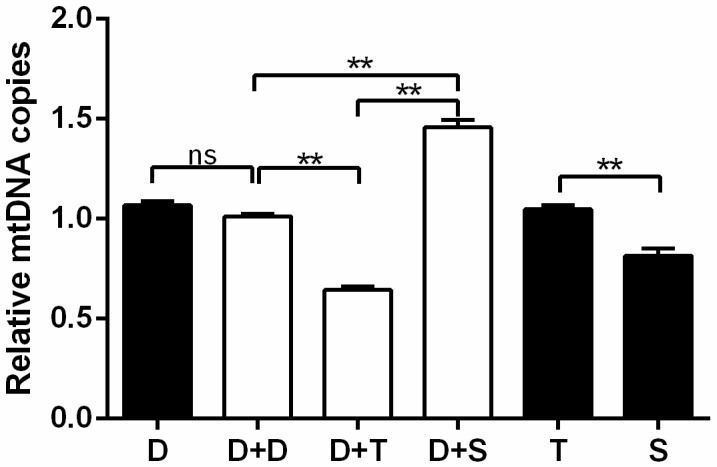
Measurement of relative mtDNA copies in primary cells and cybrids. Black column, DF-1 cells and primary cells; white columns denote cybrids. *n* = 3 per group. ** means *p* < 0.01 and ns means *p* > 0.05.

mtDNA copy number of primary chicken cells and cybrids were detected and calculated as 2^−ΔΔCT^ according to the qPCR analysis (Figure 4). In the study, the primary Tibetan chicken embryo cells (T) carried higher mtDNA copies than the Shouguang chicken primary cells (S) (*p* < 0.01). However, the Tibetan chicken cybrid (D+T) harbored the lowest amounts of mtDNA among all cybrids (*p* < 0.01). However, Shouguang cybrid (D+S) had a significantly higher amount of mtDNA than D+D (*p* < 0.01). 

### 3.5. Expression Levels of Genes Involved in Mito-Biogenesis and Mito-Fusion

**Figure 5:**
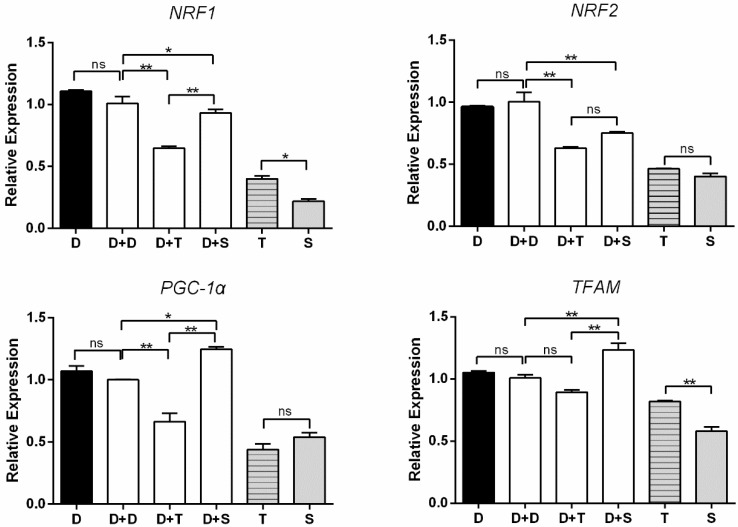
Expression levels of four mitochondrial biogenesis-related genes in primary cells and cybrids. Black column: DF-1 cells and primary cells; white columns denote cybrids. *n* = 3 per group. * means *p* < 0.05, ** means *p* < 0.01, and ns means *p* > 0.05.

The expression of four genes (*NRF1*, *NRF2*, *TFAM*, *PGC1-α*) was used to measure mitochondrial biogenesis (Figure 5). D and D+D showed the consistent tendency of expression in the four genes. D+T exhibited lower expression levels in *NRF1* and *PGC1-α* than the other cybrids (*p* < 0.01). No significant difference was observed between D+D and D+T in *TFAM*. D+S illustrated a higher level of expression than D+D in *PGC1-α* and *TFAM* expression levels (*p* < 0.05 and *p* < 0.01, respectively). There was no significant change between D+T and D+S in *NRF2*. For primary embryonic cells, T presented higher expression levels of *NRF1* and *TFAM* than S (*p* < 0.05, *p* < 0.01, respectively), similar expression levels of the other two genes observed.

**Figure 6:**
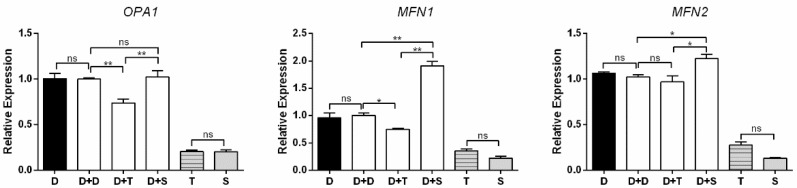
Expression levels of three mitochondrial fusion-related genes in primary cells and cybrids. Black columns: DF-1 cells and primary cells; white columns denote cybrids. *n* = 3 per group. * means *p* < 0.05, ** means *p* < 0.01, and ns means *p* > 0.05.

The expression levels of *OPA1*, *MFN1*, and *MFN2* were used to measure the mitochondrial fusion (Figure 6). Similar to the evaluated genes above, D and D+D have constant expression levels in all analyses. For cybrids, D+T showed lower expression levels of *OPA1* and *MFN1* than D+D (*p* < 0.01 and *p* < 0.05, respectively), and no significant change was observed between D+T and D+D in *MFN2*. For *MFN1* and *MFN2*, D+S had higher expression levels than D+D (*p* < 0.05 and *p* < 0.01, respectively), and a similar expression level was observed between D+S and D+D for *OPA1*. For primary cells, T presented similar expression levels to S for these three genes.

## 4. Discussion

Various mtDNA haplotypes have developed during millions of years of evolution. Previous studies have shown that the polymorphisms in mtDNA were associated with many different phenotypes, such as human disease and animal productive traits [6,8,10]. By generating cybrids, in which the mitochondria from one cell line are transferred into a ρ^0^ cell line, direct evidence can be shown to explain the association. For example, having the human mtDNA haplotype J is beneficial in decreasing the rate of incident knee osteoarthritis over time [27], while haplotype H decreases the risk for age-related macular degeneration [28]. Furthermore, the mechanism of interaction between the mitochondria and nucleus can be studied.

Compared to Shouguang chicken (S), Tibetan chicken (T) showed a higher amount of mtDNA in primary cells. Moreover, Tibetan chicken primary cells presented higher basal respiration, ATP production, and maximal respiration (Figure 2A), which allows us to infer a biological adaptation to the harsh environment and resistance to the cold condition. Tibetan chicken cells exhibited higher glycolysis and glycolysis capacity (Figure 3A). A previous study had already suggested that the Tibetan chicken has higher glucose oxidation, mitochondrial metabolism, and antioxidant ability [29]. Combined with our results, the huge differences seen between the Tibetan chicken and Shouguang chicken indicate an adaptation to the hypoxic environment of the Tibetan chicken. 

Compared to other chicken cybrids (D+D, D+S), the cybrid from the Tibetan chicken (D+T) manifested generally lower OCR indexes (basal respiration, ATP production, and maximal respiration), mtDNA copy number, and mRNA expression of genes involved in mito-biogenesis and mito-fusion, but showed a higher ECAR index (glycolysis and glycolysis reserve). Tibetan chicken primary cells (T) exhibited a higher rate of ATP production than others, since higher mtDNA copies were detected in Tibetan chicken primary cells (Figure 4). However, the metabolic level per mitochondrion in Tibetan chicken primary cells was similar to the mitochondrion of Shouguang chicken primary cells based on the mitochondrial content differences. However, Tibetan chicken cybrid appeared “weaker” in mitochondrial metabolism. So, to keep normal physical activities, the cybrid produced the ATP through glycolysis as a compensatory mechanism, and that may be the reason the Tibetan cybrid (D+T) harbored a similar ECAR to the Shouguang cybrid (D+S) (Figure 3B). Due to most proteins in the five complexes of the electron transfer chain being encoded by nuclear DNA and then transferred into the mitochondrion, it is required that mitochondria maintain coordinated communication with the nucleus [30]. The DF-1 cell line was derived from the East Lansing Line, a lowland breed. And the alignment D/S only had two missense values that are moderately conserved, whereas the alignment D/T had four missense values that are moderately conserved (Table 1). It indicated that the combination of the D nucleus and T mtDNA haplotype was more divergent than the Shouguang cybrids (D+S). These results support the hypothesis of mitochondria–nuclear incompatibility and mitochondria–nuclear coevolution [31,32].

*NRF1*, *NRF2*, *TFAM*, and *PGC-1α* are involved in the critical pathway of mitochondrial biogenesis and metabolic processes [33,34]. *PGC-1α* is an essential transcriptional coactivator in inducing mitochondrial biogenesis by activating nuclear respiratory factor 1 (*NRF1*), nuclear respiratory factor 2 (*NRF2*), and other transcription factors. *NRF1* and *NRF2* can activate mitochondrial transcription factor A (*TFAM*) to modulate the mitochondrial activity [33]. *OPA1*, *MFN*, and *MFN2* are mito-fusion genes. Specifically, *OPA1* mediate the fusion of the inner membrane, and *MFN1* and *MFN2* modulate the fusion of the outer membrane. The fusion of mitochondria induces supercomplexes of the ETC, maximizing OXPHOS activities [35,36]. In this study, among these cybrids, the Shouguang cybrid (D+S) harbored higher expression levels of these genes (Figure 5 and Figure 6). Meanwhile, D+S exhibited a generally higher OCR than other cybrids, with higher basal respiration, ATP production, and reserve capacity (Figure 2B), reflecting an active energy metabolism. Since all these genes are encoded by the nuclear genome, the results suggest that the expression of these genes could be regulated by the mtDNA haplotype. Previous studies also showed that with the same nuclear genome, the mtDNA haplotype can regulate the transcription of the nuclear genes by methylation [37]. These results suggested that haplotype S could communicate more effectively with the nucleus of the DF-1 cell than haplotype T and D. Moreover, a broad range of interactions between the genomes does not only depend on the subunit of protein complexes but also rely on the protein/RNA and protein/DNA interplay [38].

## 5. Conclusions

In conclusion, our study provides the first evidence that under the same nuclear background, specific mtDNA haplotypes can induce functional differences in cellular energy metabolism in *Gallus gallus domesticus*. Our findings explain the associations between the mtDNA variants and specific traits, and may give insight into the effects of mtDNA haplotypes on the metabolic phenotypes of farm animals. 

## Figures and Tables

**Table 1:** Mitogenome divergences among the mitochondrial haplotypes.

Comparison	SNPs/InDels					AA Change	Grantham Score Summary
	D-loop	rRNA	tRNA	Protein	Total		
D/T	9	2	1	26	39	5	1 in 0–50, 4 in 51–100
D/S	10	4	2	29	46	4	2 in 0–50, 2 in 51–100
T/S	11	4	1	25	41	5	1 in 0–50, 4 in 51–100

## Supplementary Materials

The following are available online at https://www.mdpi.com/2073-4425/11/1/100/s1, Table S1: Primer pairs for the amplification of chicken mitochondrial DNA sequences, Table S2: Primer pairs for real-time PCR, Table S3: Detailed variant sites among three chicken mitogenomes, Table S4: Detailed information of missense mutations.
