# Supplementary material for: Mitochondrial DNA Haplotypes Influence Energy Metabolism across Chicken Transmitochondrial Cybrids
Source: Genes (Basel). 2020 Jan 16;11(1):100. doi: 10.3390/genes11010100 (PMC7017162; doi:10.3390/genes11010100)
Supplement: Supplementary file 1 [file genes-11-00100-s001.zip › Table S1.docx]

**Table S1. Primer pairs for the amplification of chicken mitochondrial DNA sequences**

| Primer  Pair No. | Primer sequence (5’ - 3’) | Position* | Expectant size(bp) |
| --- | --- | --- | --- |
| 1 | F: CTCGCCCTACTTGCCTTCC | 427-445 | 925 |
|  | R: TGCCTGATACCTGCTCCTTT | 1351-1332 |  |
| 2 | F: CACTGAAGATGCCAAGATGGTA | 1192-1213 | 748 |
|  | R: CCTTGACCTGTCTTATTAGCGA | 1939-1918 |  |
| 3 | F: TGCCAGCACAGCCTACATA | 1845-1863 | 750 |
|  | R: GAGACGGGTTCGCTCAAAT | 2594-2576 |  |
| 4 | F: TAGCAAGAACAACCAAGCAAAGTG | 2500-2523 | 782 |
|  | R: CCATTCATACAAGTCTCAATTTAC | 3281-3258 |  |
| 5 | F: GCAAACCAAAGACCCGACTG | 3117-3136 | 898 |
|  | R: GAAGGTTTGTTAGGGTGGG | 4014-3996 |  |
| 6 | F: TTAACAGTCCTACGTGATCTGAGT | 3708-3731 | 1077 |
|  | R: GAGTGCAATGGGAAATAATTCT | 4784-4763 |  |
| 7 | F: TAACCACCGTCCTATTCCTG | 4717-4736 | 699 |
|  | R: ATCAGGCGTTGGTTATGCT | 5415-5397 |  |
| 8 | F: CGAGCGATTGAAGCCACTA | 5328-5346 | 740 |
|  | R: GCAAGTCGGAGGTAGAAGAAT | 6067-6047 |  |
| 9 | F: GGCTTCATGCCAAAATGACT | 5952-5971 | 1109 |
|  | R: AGAATGGAGGAAACACCTGCTA | 7060-7039 |  |
| 10 | F: CCTACTAGCCTCATCTACCGTAG | 6917-6939 | 1005 |
|  | R: GCGTCTGGGTAATCTGAGTATC | 7921-7900 |  |
| 11 | F: ACAGGCTTTACCCTACACCCA | 7785-7805 | 1172 |
|  | R: GGTTAAGATGACAGTAGTGAGG | 8956-8935 |  |
| 12 | F: GCAATCCCTGGACGACTAAATCA | 8787-8809 | 1122 |
|  | R: ATGGGCTTGGGTCAACTATGTG | 9908-9887 |  |
| 13 | F: ACCAATAATACCATCAATCTCC | 9728-9749 | 1034 |
|  | R: CGCTTAGTAGAAAGGATAGTGAG | 10761-10739 |  |
| 14 | F: TTTGCCTCCTACGACTAATCAA | 10512-10533 | 1008 |
|  | R: GCTGTATATTGTGGTGTTAGTTC | 11519-11497 |  |
| 15 | F: TCATTCGCCCTTGGACCTAT | 11369-11388 | 682 |
|  | R: TTGGGGTGGGTGAGTTTGAT | 12050-12031 |  |
| 16 | F: CATTCGCCCTTGGACCTATC | 11370-11389 | 1388 |
|  | R: GATGGAAGAGTGCCTCGTTGG | 12758-12738 |  |
| 17 | F: CCTAAAATCCCTCATTGCCTAC | 12269-12290 | 1157 |
|  | R: TATGTTATTTGCGATGGTTAGTG | 13425-13403 |  |
| 18 | F: GAAAGCATTGCCACCCACTGA | 13192-13212 | 1141 |
|  | R: TGATTGCTGGGGTTCGTGTG | 14332-14313 |  |
| 19 | F: CCACCTCCTGCCTAACCATT | 14117-14136 | 1020 |
|  | R: TCGTCCGATGTGAAGGAAGATA | 15136-15115 |  |
| 20 | F: AACGTACAATACGGCTGAC | 15050-15068 | 1022 |
|  | R: AGGTTTGAGTCCTCCTTTT | 16071-16053 |  |
| 21 | F: CCCCACAATCGGAACACTA | 15934-15952 | 735 |
|  | R: GGTCTAACCAAGCGGGAATA | 16668-16649 |  |
| 22 | F: GATTAGACGCCACAGCTAAA | 16650-16669 | 721 |
|  | R: TTCGTGAAAAGTGAGAAAGTTC | 584-563 |  |

*Positions are relative to the KM433666 sequence.
