# Supplementary material for: Mitochondrial DNA Haplotypes Influence Energy Metabolism across Chicken Transmitochondrial Cybrids
Source: Genes (Basel). 2020 Jan 16;11(1):100. doi: 10.3390/genes11010100 (PMC7017162; doi:10.3390/genes11010100)
Supplement: Supplementary file 1 [file genes-11-00100-s001.zip › Table S2.docx]

**Table S2. Primer pairs for real-time PCR**

| Gene | Primer sequence (5’ - 3’) | Product size (bp) | Accession number |
| --- | --- | --- | --- |
| *18S* | F: ATAACGAACGAGACTCTGGCA | 136 | AF173612.1 |
|  | R: CGGACATCTAAGGGCATCACA |  |  |
| *ND4* | F: CCAACCACCAACCTGATAGC | 157 | KM433666.1 |
|  | R: TGTGGGATGGAAGAGTGCC |  |  |
| *NRF1* | F: TGGCACTTAACAGTGAAGCA | 174 | XM_015282769.2 |
|  | R: CGGTCTGGTACATGCTGAC |  |  |
| *NRF2* | F: ACGGTGACACAGGAACAACA | 279 | NM_205117.1 |
|  | R: GCACACTCCCAGGAGAACTG |  |  |
| *PGC-1α* | F: CATCTCCAGCCAGTACAGCA | 177 | NM_001006457.1 |
|  | R: GCGTCATGTTCATTGGTCAC |  |  |
| *Tfam* | F: CCAGCAGAACCCAGAACTGA | 146 | NM_204100.1 |
|  | F: TGTATGCAGCCAACTGCTCT |  |  |
| *OPA1* | F: TCCTGATGCCGATGACCTTG | 126 | XM_015291400.2 |
|  | R: TGGTCCGTAGCTCTGAATGC |  |  |
| *MFN1* | F: ATCGTTGTTGGCGGAGTGAT | 160 | NM_001012931.2 |
|  | R: TTTCGGTCGCGTAGTTCACA |  |  |
| *MFN2* | F: AGAGCAAAGCAGATCGCAGA | 242 | XM_015297206.2 |
|  | R: AGCACTGAAAGCCGTCTGAT |  |  |
| *β-actin* | F: GATATTGCTGCGCTCGTTGT | 152 | NM_205518 |
|  | R: AACCATCACACCCTGATGTCT |  |  |
