# Supplementary material for: Mitochondrial DNA Haplotypes Influence Energy Metabolism across Chicken Transmitochondrial Cybrids
Source: Genes (Basel). 2020 Jan 16;11(1):100. doi: 10.3390/genes11010100 (PMC7017162; doi:10.3390/genes11010100)
Supplement: Supplementary file 1 [file genes-11-00100-s001.zip › Table S3.docx]

**Table S3. Detailed variant sites among three chicken mitogenomes**

| Position | D (MK163563) | T (MK163562) | S (MK163561) | Region |
| --- | --- | --- | --- | --- |
| 167 | T | T | C | D-loop |
| 199 | C | T | T | D-loop |
| 217 | C | T | T | D-loop |
| 225 | C | C | T | D-loop |
| 243 | C | C | T | D-loop |
| 256 | C | C | T | D-loop |
| 261 | T | T | C | D-loop |
| 281 | A | G | A | D-loop |
| 306 | T | C | T | D-loop |
| 310 | T | T | C | D-loop |
| 355 | T | C | T | D-loop |
| 391 | C | T | C | D-loop |
| 446 | T | C | C | D-loop |
| 852 | - | C | - | D-loop |
| 1214 | T | C | C | D-loop |
| 2071 | C | C | T | 12S |
| 2139 | G | G | A | 12S |
| 2595 | G | A | G | 16S |
| 3837 | A | A | G | 16S |
| 3941 | C | T | T | 16S |
| 4586 | A | G | G | ND1 |
| 4628 | A | A | G | ND1 |
| 5724 | T | G | T | ND2 |
| 5870 | C | T | C | ND2 |
| 6764 | C | T | T | COX1 |
| 6806 | C | T | T | COX1 |
| 6905 | G | A | A | COX1 |
| 8079 | C | T | T | COX1 |
| 8339 | C | T | T | / |
| 8473 | C | T | T | COX2 |
| 8618 | C | T | T | COX2 |
| 9162 | C | T | T | ATP8 |
| 9467 | C | T | C | ATP6 |
| 9542 | G | A | A | ATP6 |
| 9602 | A | A | G | ATP6 |
| 9806 | A | A | G | ATP6 |
| 9863 | G | A | G | ATP6 |
| 10081 | G | A | A | COX3 |
| 10447 | C | C | T | COX3 |
| 10978 | C | T | T | ND3 |
| 11007 | C | C | T | ND3 |
| 11374 | G | A | G | ND4L |
| 11388 | T | C | C | ND4L |
| 11693 | A | G | A | ND4 |
| 11973 | T | C | C | ND4 |
| 12104 | C | C | T | ND4 |
| 12377 | G | G | A | ND4 |
| 12464 | C | T | T | ND4 |
| 12689 | C | C | T | ND4 |
| 12961 | G | G | A | tRNA-Ser |
| 13229 | T | T | C | ND5 |
| 14707 | C | A | C | ND5 |
| 14877 | G | C | G | ND5 |
| 15235 | G | A | G | CytB |
| 15379 | C | T | C | CytB |
| 15409 | T | C | T | CytB |
| 15445 | T | C | C | CytB |
| 16131 | G | A | A | tRNA-Pro |
| 16263 | C | C | T | ND6 |
| 16371 | C | C | T | ND6 |
| 16491 | C | C | T | ND6 |
| 16596 | G | G | A | ND6 |
| 16607 | C | C | T | ND6 |
