# Supplementary material for: Mitochondrial DNA Haplotypes Influence Energy Metabolism across Chicken Transmitochondrial Cybrids
Source: Genes (Basel). 2020 Jan 16;11(1):100. doi: 10.3390/genes11010100 (PMC7017162; doi:10.3390/genes11010100)
Supplement: Supplementary file 1 [file genes-11-00100-s001.zip › Table S4.docx]

**Table S4. Detailed information of missense mutations**

| Gene | Nucleotide position | Nucleotide change | | | AA changes | Grantham Score |
| --- | --- | --- | --- | --- | --- | --- |
|  |  | D (MK163563) | T (MK163562) | S (MK163561) |  |  |
| ND2 | 5724 | T | G | T | S160A | 99 |
| COX1 | 8079 | C | T | T | L477F | 22 |
| COX2 | 8473 | C | T | T | T45M | 81 |
| ND4L | 11374 | G | A | G | A57T | 58 |
| ND5 | 13229 | T | T | C | I50T | 89 |
| ND5 | 14707 | C | A | C | H543N | 68 |
| ND6 | 16607 | C | C | T | V37M | 21 |
